# Use of cerebral state index to predict long-term unconsciousness in patients after elective craniotomy with delay recovery

**DOI:** 10.1186/1471-2377-11-15

**Published:** 2011-01-27

**Authors:** Ming Xu, Yan-Ni Lei, Jian-Xin Zhou

**Affiliations:** 1 Department of Critical Care Medicine, Beijing Tiantan Hospital, Capital Medical University, No 6, Tiantan Xili, Chongwenqu, Beijing, 100050, China

## Abstract

**Background:**

The major difficulty in postoperative care in patients after craniotomy is to distinguish the intracranial deficits from the residual effect of general anesthesia. In present study, we used cerebral state index (CSI) monitoring in patients after craniotomy with delayed recovery, and evaluated the prediction probability of CSI for long-term postoperative unconsciousness.

**Methods:**

We enrolled 57 consecutive adult patients admitted to neurosurgical intensive care unit (NICU) after elective craniotomy with delayed recovery. CSI was continuously monitored for 6 hours after admission. Patient's level of consciousness was followed up for 24 hours. According to whether obeyed verbal command, patients were divided into awaken group and non-awaken group. CSI values were compared between the two groups. Prediction probability (P_K_) was calculated to determine the probability of CSI in predicting unconsciousness 24 hours after operation.

**Results:**

In awaken group (n = 51), CSI increased significantly after the 2nd NICU admitted hour (*P *< 0.05). At each time point, CSI values in awaken group were significantly higher than those in non-awaken group (n = 6) (*P *< 0.05). The values of P_K _(SE) for CSI in the first 6 admitted hours ranged from 0.94 (0.06) to 0.99 (0.02).

**Conclusions:**

In patients after craniotomy with delayed recovery, CSI monitoring in early postoperative hours had high prediction probability for long-term unconsciousness. CSI monitoring may be a reliable objective method to predict level of consciousness after elective craniotomy.

## Background

The most feared complications after craniotomy are formation of intracranial hematoma and major brain swelling. Although rapid emergence from general anesthesia is desirable in the majority of neurosurgical patients, in certain cases whose systemic or brain homeostasis is impaired, delayed recovery may be a better choice [1]. However, delayed recovery usually prevents the timely diagnosis of cerebral complications after craniotomy. Therefore, one of the main issues in postoperative care in delayed recovery is to distinguish the unresponsive state that is indicative of intracranial reasons from the residual effects of general anesthesia [1]. In clinical practice, evaluation of consciousness is largely based on subjective neurological examination, such as Glasgow Coma Scale and pupil size and reaction to light. Although many efforts have been made, it is still difficult to measure consciousness by objective instruments.

In order to monitor the depth of anesthesia objectively and quantitatively, several processed electroencephalogram (EEG) algorithms have been designed and studied extensively in operating room, but to a much lesser degree, in postoperative care and brain injury [2]. In 2004, the cerebral state index (CSI) monitor (Danmeter, Odense, Denmark) was launched as a new processed EEG monitor for measuring hypnotic depth [3]. Up to now, clinical studies of CSI monitoring in postoperative care are limited, especially for neurosurgical patients.

In present study, CSI monitoring was used in patients after elective craniotomy with delayed recovery from general anesthesia. The aim of this study was to test whether CSI monitoring in early postoperative recovery hours could predict long-term unconsciousness in advance.

## Methods

The study protocol was reviewed and approved by Research Ethic Committee in Beijing Tiantan Hospital, Capital Medical University (Beijing, China). Written informed consent was obtained from patients or their healthcare surrogates.

The study was carried out in a neurosurgical intensive care unit (NICU) of a 1000-bed university hospital over a 3 month period, from November 2007 to January 2008. Our NICU is open to neurosurgical patients 24 hrs per day, 7 days per week, and all craniotomy patients are admitted to NICU for postoperative care. During the study, routine practices of anesthesia and postoperative care were followed, and no attempt was made to change or influence the standard practices.

In our hospital, all craniotomy were performed under general anesthesia. Typically, anesthesia was induced with propofol, 2 mg/kg IV, and sufentanil, 1-2 μg/kg IV, and tracheal intubation was facilitated with vecuronium, 0.1 mg/kg IV. Anesthesia was maintained by sevoflurane in oxygen (with end-tidal concentration of 1.5%-2%), and sufentanil (infusion rate of 0.2-0.5 μg/kg/hr), titrated to keep mean blood pressure within 30% of preoperative values. Vecuronium (bolus of 0.05 mg/kg) was administered according to train of four monitoring. At the time of dural closure, sufentanil infusion was discontinued. Sevoflurane was discontinued during skin closure. Anesthesiologists and Neurosurgeons discussed patient's status, and made the decision of recovery. Typically, delayed recovery was scheduled in patients with following conditions: 1) emergency craniotomy in traumatic brain injury and intracranial hematoma; 2) large or complicated arterio-venous malformation resection; 3) large tumor resection with preoperative midline shift; 4) major intraoperative bleeding or brain swelling; 5) extensive posterior fossa operation involving cranial nerves IX-XII; 6) impaired preoperative state of consciousness; 7) intraoperative abnormal body temperature, inadequate oxygenation, cardiovascular instability, or coagulation disorder; 8) length of operation longer than 6 hours.

For delayed recovery, patient was remained tracheal intubated at the end of surgery and transported to NICU with manual ventilation and supplemental oxygen. On arrival to the NICU, mechanical ventilation and standard clinical monitoring devices were applied (including 5-lead continuous electrocardiogram, pulse oximeter, noninvasive blood pressure, capnograph, and rectal temperature). All patients were warmed during NICU stay by using a forced-air warming blanket to maintain rectal temperature above 36°C. Criteria for tracheal extubation included: 1) obey verbal command; 2) have adequate spontaneous breathing and oxygenation; 3) possess an intact gag reflex. All extubated patients were given supplemental oxygen by mask. Postoperative sedation was not used deliberately. In patients who exhibited agitation but did not meet the extubation criteria, midazolam was IV infused for 2 hours (0.05-0.2 mg/kg/hr IV). The dose of midazolam was titrated to obtain light sedation (patient unresponsive to verbal command, but showing motor response to noxious stimuli). Arterial blood gas, serum electrolytes, whole blood counts, blood urea nitrogen, and blood glucose analysis were performed during the first 2 postoperative hours. Postoperative computed tomographic scan was not used routinely, but was usually carried out in patients who exhibited unexplained delayed awakening or new neurological deficits.

During study period, adult patients (> 18 yrs of age) after elective craniotomy with delayed recovery were enrolled consecutively, excluding those with impaired preoperative level of consciousness. Demographic data were collected at patient's admission, which included age, sex, and length of operation. CSI monitoring was set up within 30 min after patient's arrival in NICU. The skin was prepared by swabbing with alcohol and then firmly rubbing with abrasive paper. Standard wet gel ECG electrodes (SKINTACT^®^, Leonhard Lang GmbH, Innsbruck, Austria) were applied according to the manufacturer's instruction, with one on the forehead's midline, one more laterally on the forehead, and one on the mastoid process behind the ear. Electrodes were attached to a handheld CSI monitor (Danmeter, Odense, Denmark, SN 2006219982) by a snap connector. After an initial control of electrode impedance, the monitor displayed a numerical CSI from 0 to 100. CSI was continuously monitored for 6 hours after NICU admission. Glasgow coma scale was assessed every 1 hour for 6 hours. Because all enrolled patients had endotracheal intubation at entry of the study, we only documented the motor responses to external stimuli in Glasgow coma scale (GCS-M) [4]. Patients were given a verbal command to open eyes or lift hands, first in a normal voice, and then in a loud voice. If patients did not respond to the loud verbal command, they were given a light tap on the shoulder and verbal command simultaneously. Obeying command in response to the shoulder tap and verbal command was considered as a positive response (GCS-M = 6). If there was still no response, a painful stimulus of rubbing the sternum was applied to differentiate pain localization (GCS-M = 5), withdrawal flexion (GCS-M = 4), stereotyped flexion (GCS-M = 3), stereotyped extension (GCS-M = 2), and none response (GCS-M = 1). Immediately after each hour's verbal or painful stimulation in GCS-M evaluation, CSI value was observed for 2 min by a nurse (not involved in this study), and the maximal value was documented manually. Use of sedatives during the CSI monitoring was also recorded.

Patients were followed up at 24-hour after the end of surgery, and level of consciousness was evaluated. In patients under sedation, sedatives were stopped for at least 1 hr to facilitate a reliable neurological assessment. According to the results of follow-up, patients were divided into two groups: awaken group (obey verbal command evaluated as GCS-M = 6) and non-awaken (GCS-M = 5 to 1).

Continuous variables (CSI, age, and length of operation) were expressed as mean and SD, and ordinal variables (GCS-M) were expressed as median and interquartile range (IQR). For CSI and GCS-M data, within-group comparisons across time points were performed by non-repeated-measures of analysis of variance with a *post hoc *Student-Newman-Keuls multiple comparison test, and between-group comparisons in each time points were performed by unpaired Student's *t *test for CSI and the Wilcoxon rank sum test for GCS-M. Categorical variables were expressed as numbers and percentages, and χ^2 ^test was used for comparison between the two groups. Spearman rank-order correlation analysis was used to evaluate the relationship between GCS-M and CSI. For evaluating the probability of CSI in predicting unconsciousness 24 hrs after operation, we calculated the prediction probability (P_K_) as described by Smith et al [5]. P_K _was calculated as the Somers' *d *statistic using SPSS version 10.0, which was then transformed from -1 to +1 range of Somers' *d *to 0 to 1 range of P_K _by using the equation [6]:

PK=1−(1−|Somers'd|)/2

Standard error (SE) of P_K _was calculated as the SE of Somers' *d *divided by 2. Best-fitting logistic curve between CSI and the probability of unconsciousness was plotted. The values of CSI associated with a probability of 50% and 95% for unconsciousness at 24-hour after operation (CSI_50% _and CSI_95%_) were estimated by using logistic regression analysis, and 95% confidence limit (95% CL) was calculated.

Statistical analysis was carried out by SPSS version 10.0 (SPSS, Chicago, IL, USA). A *P*-value less than 0.05 was considered statistically significant.

## Results

During the study period, 487 adult patients after elective craniotomy were admitted in our NICU for postoperative care. Among these patients, 62 were treated with delayed recovery, of whom 57 were enrolled and 5 were excluded due to decreased level of consciousness preoperatively. Fifty-one patients obeyed verbal command at the 24-hour postoperative follow-up (awaken group), and 6 patients did not (non-awaken group). The patients in the 2 groups were comparable as to demographic characteristics (Table 1).

**Table 1:** Demographic characteristics of the patients

	Awaken group (n = 51)	Non-awaken group (n = 6)	*P*
Age, yrs, mean ± SD	45 ± 15	47 ± 18	0.75
Males, n (%)	30 (59%)	4 (67%)	0.71
Length of operation, min, mean ± SD	371 ± 84	356 ± 77	0.67
Sedative use during the first 6 hrs, n (%)	11 (22%)	1 (17%)	0.78

**Figure 1:**
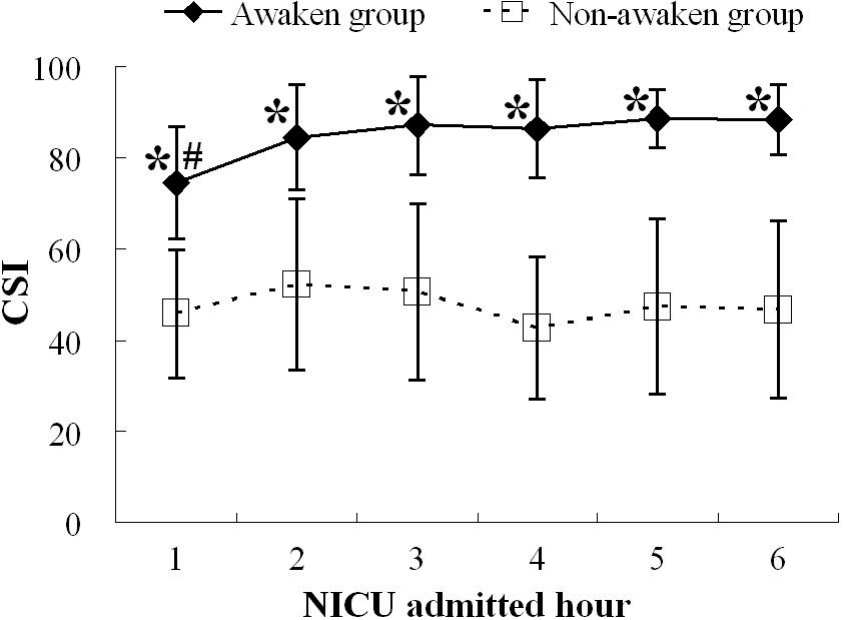
**Cerebral state index (CSI) at the first to 6th neurosurgical intensive care unit (NICU) admitted hours in awaken and non-awaken group**. * *P *< 0.05 between the groups; # *P *< 0.05 compared to values at 2nd to 6th NICU admitted hour.

Figure 1 showed CSI data in the two groups for the first 6 NICU admitted hours. In awaken group, CSI value at the 1st hour of admission was 75 ± 12. CSI increased significantly at the 2nd admitted hour (*P *< 0.05), but there was no significant changes during the 2nd to 6th hours (85 ± 12 to 88 ± 7). There was no significant change of CSI across different time points in non-awaken group, ranging from 43 ± 15 to 52 ± 19 (*P *> 0.05). Inter-patient variability of CSI existed in each group, which was demonstrated by a significant difference in random factor of patient in non-repeated-measures of analysis of variance (*P *< 0.05). At each time point, CSI values in non-awaken group were significantly lower than those in awaken group (*P *< 0.05, Figure 1).

GCS-M data were shown in Table 2. GCS-M increased significantly during the early NICU admitted hours. GCS-M in awaken group were significantly higher than those in non-awaken group (*P *< 0.05). At the first NICU admitted hour, no patient obeyed verbal command in either group. At the 6th admitted hour, 88% of patients in awaken group obeyed verbal command, but still no patient obeyed in non-awaken group (Table 2).

**Table 2:** GCS-M data and number of patients obeying verbal commands.

NICU admitted hours	GCS-M, median (IQR)		Number of patients obeying verbal commands, n/N (%)	
	
	Awaken group (n = 51)	Non-awaken group (n = 6)	Awaken group (n = 51)	Non-awaken group (n = 6)
1	4 (4-5) *|	2 (1-2)	0/51 (0)	0 (0)
2	5 (4-6) *†	2 (1-3)	17/51 (33%)*	0 (0)
3	6 (5-6) *‡	2 (1-3)	30/51 (59%)*	0 (0)
4	6 (5-6) *	2 (1-3)	37/51 (73%)*	0 (0)
5	6 (6-6) *	2 (1-3)	45/51 (88%)*	0 (0)
6	6 (6-6) *	2 (1-3)	45/51 (88%)*	0 (0)

GCS-M: the motor response to external stimuli in Glasgow Coma Scale; IQR: interquartile range; NICU: neurosurgical intensive care unit.

* *P *< 0.05 between the groups; | *P *< 0.05 compared to values of 2nd to 6th admitted hour; † *P *< 0.05 compared to values of 3rd to 6th admitted hour; ‡ *P *< 0.05 compared to values of 5th and 6th admitted hour.

**Figure 2:**
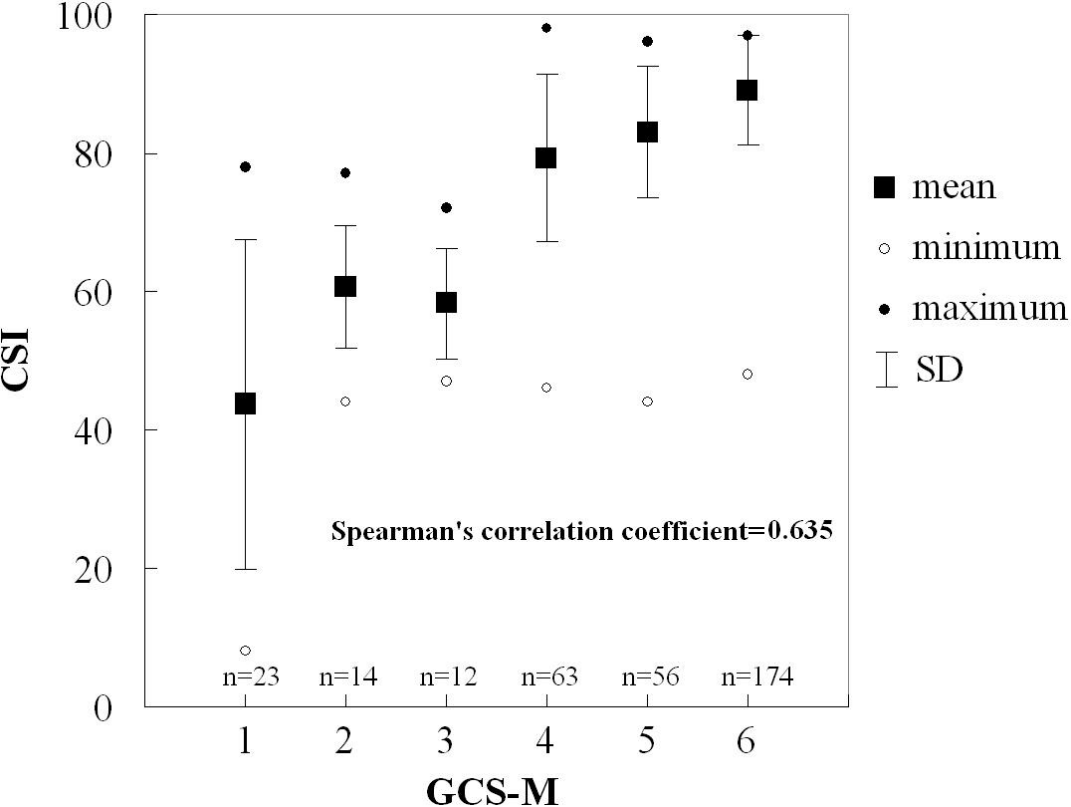
**Correlation between the motor response to external stimuli in Glasgow Coma Scale (GCS-M) and cerebral state index (CSI)**. To demonstrate the scatter of data, mean, standard deviation (SD), the minimum, and the maximum values of CSI are presented. Numbers of data sets are also presented. Spearman's correlation coefficient was calculated from all individual data set.

A significant correlation was found between CSI and GCS-M from all data sets (Spearman's correlation coefficient = 0.635, *P *< 0.05, Figure 2).

The values of P_K _(SE) for CSI in the first 6 NICU admitted hours ranged from 0.94 (0.06) to 0.99 (0.02) (Table 3). CSI_50% _and CSI_95% _were also shown in Table 3.

**Table 3:** P_K_, CSI_50% _and CSI_95% _calculated from different time point data.

NICU admitted hours	PK (SE)	CSI_50% _(95% CL)	CSI_95% _(95% CL)
1	0.94 (0.06)	43 (18-53)	18 (-48-34)
2	0.95 (0.05)	54 (25-64)	30 (-45-46)
3	0.97 (0.04)	56 (31-66)	32 (-31-48)
4	0.99 (0.02)	55 (9-64)	42 (-18-51)
5	0.99 (0.03)	63 (50-87)	53 (-57-60)
6	0.99 (0.02)	63 (44-72)	49 (-1-59)

P_K_: prediction probability; CSI_50% _and CSI_95%_: the values of cerebral state index associated with a probability of 50% and 95% for unconsciousness 24 hr after craniotomy; NICU: neurosurgical intensive care unit.

## Discussion

In present study, CSI monitoring was used in early postoperative period in craniotomy patients with delayed recovery. The main result of our study is that CSI values were significantly different between patients who awake and those who remained unconscious at 24-hour after operation, and that CSI monitoring in early postoperative hours had high prediction probability for long-term postoperative unconsciousness.

The major difficulty in postoperative care in patients after craniotomy, especially for the situation of delayed recovery, is to distinguish the intracranial deficits from the residual effect of general anaesthesia [1]. Although the bedside physical examination is the standard method of assessment of consciousness in neurosurgical patients, EEG is an objective tool that permits sensitive and continuous monitoring of brain function. However, because interpretation of the raw EEG signal requires considerable expertise and specialised training, more standardised and simpler measures of brain function are desirable [7]. In order to measure the depth of hypnotic objectively and quantitatively, several processed EEG algorithms, such as the bispectral index (BIS) [8], the Narcotrend [9], the SNAP index [10], and more recently, CSI [3,11], are designed. The CSI value is passively derived from EEG signals and provides a dimensionless number from 0 to 100. It uses a fuzzy logic inference system based on power analysis of beta, alpha and beta-alpha ratio with an estimation of burst suppression ratio. Recent attempts have been made to extend the use of BIS to brain-injured patients [12,13], and the results of these studies indicate that BIS value correlates with the severity of brain-injury. In present study, we enrolled craniotomy patients with delayed recovery, and performed CSI monitoring in early postoperative period. The patient's level of consciousness was followed up at 24-hour after craniotomy to excluding the residual effect of general anaesthesia. By this study design, we could evaluate the predicting ability of early postoperative CSI monitoring to long-term neurological outcome. Early postoperative CSI values in patients with long-term neurological deficits were significantly lower than those recovered (Figure 1). The CSI values in the first 6 NICU admitted hours ranged from 75 to 88 in awaken group, and 43 to 52 in non-awaken group. For patients in awaken group, CSI values remained relatively stable after the 2nd NICU admitted hour (Figure 1). Further more, the P_K _statistic analysis showed the good predictive ability of CSI for detecting long-term postoperative unconsciousness (Table 3). From 2nd to 6th NICU admitted hours, the CSI values associated with probability of 50% and 95% for long-term unconsciousness after craniotomy, which we named as CSI_50% _and CSI_95%_, were 54 to 63 and 30 to 53, respectively (Table 3). Based on these results, it can be said that those patients whose CSI values are lower than 54 to 63 in the first 6 postoperative hours have a probability of long-term postoperative unconsciousness higher than 50%, and those with CSI values lower than 30 to 53 have a probability higher than 95%. If CSI monitoring shows a value below these borderlines, physician should exam the patient carefully to exclude the intracranial complications.

There are some limitations in our study. First, we did not computerize CSI values recording, and therefore our results may not accurately reflect rapid changes. But the lack of such fast CSI data extractions may not affect the clinical question, because majority of patients in our study were relatively stable during the stay in NICU. Second, because there was no attempt made to change the standard practices, we did not control postoperative sedation in our study. Midazolam was used in 11 and 1 patients in awaken and non-awaken group, respectively. When patients in awaken group were stratified by the use of midazolam, it was interesting to find that CSI values in patients under sedation were not significantly different to those not receiving sedatives, except for the values in the 1st admitted hour (Table 4). After excluding the data from patients under postoperative sedation, P_K _values for predicting long-term unconsciousness after craniotomy were still greater than 0.94 (data are not shown). Several studies have carried out in intensive care unit patients under sedation, and results showed that the verbal and physical stimulations increased patient wakefulness with an accompanying increase in BIS values [14,15]. In our previous study, we found that CSI values increased significantly after verbal or painful stimulation, and CSI values after external stimulations were more reliable than random baseline values for detecting purposeful movement in response to external stimuli in brain-injured patients [16]. In present study, the dose of midazolam was titrated to obtain a light sedation level, and we recorded the maximal CSI values immediately after verbal or painful stimulation in GCS-M evaluation. These external stimulations might increase the CSI values. Third, we did not deliberately select the side of CSI electrode placement according to the location of operation. Previous study has found a very high correlation in CSI derived simultaneously from the left and right sides of the brain in patients without brain injury [17]. However, further studies are necessary to determine the agreement in CSI readings between the two sides in patients after craniotomy.

**Table 4:** CSI values (mean ± SD) in patients under sedation or not.

NICU admitted hours	Under sedation (n = 11)	Not receiving sedatives (n = 40)	*P*
1	63 ± 15	78 ± 9	< 0.001
2	85 ± 9	85 ± 12	0.99
3	90 ± 7	86 ± 12	0.34
4	89 ± 8	86 ± 11	0.37
5	89 ± 7	88 ± 7	0.63
6	90 ± 5	88 ± 8	0.36

CSI: cerebral state index; NICU: neurosurgical intensive care unit.

## Conclusions

Results from present study suggest that CSI correlated with postoperative unconsciousness in patients after elective craniotomy with delayed recovery. More importantly, CSI monitoring in early postoperative period had high prediction probability for long-term postoperative unconsciousness. These results would encourage conducting clinical trials in greater populations.

## Abbreviations

BIS: bispectral index; CSI: cerebral state index; EEG: electroencephalogram; GCS-M: motor responses to external stimuli in Glasgow coma scale; IQR: interquartile range; P_K_: prediction probability; NICU: neurosurgical intensive care unit;

## Competing interests

The authors declare that they have no competing interests.

## Authors' contributions

MX and JXZ contributed to the study conception and design. MX and YNL participated in recruitment of patients, performing CSI monitoring and assessing Glasgow Coma Scale. YNL participated patients' follow-up. YNL and JXZ participated statistical analysis and drafting of the manuscript. All authors read and approved the final manuscript.

## Pre-publication history

The pre-publication history for this paper can be accessed here:

http://www.biomedcentral.com/1471-2377/11/15/prepub

## References

[B1] Himmelseher S Pfenninger E Anaesthetic management of neurosurgical patients Curr Opin Anaesthesiol 2001 14 483 490 10.1097/00001503-200110000-00004 17019134

[B2] Tonner PH Paris A Scholz J Monitoring consciousness in intensive care medicine Best Pract Res Clin Anaesthesiol 2006 20 191 200 10.1016/j.bpa.2005.08.011 16634425

[B3] Anderson RE Barr G Jakobsson JG Cerebral state index during anaesthetic induction: a comparative study with propofol or nitrous oxide Acta Anaesthesiol Scand 2005 49 750 753 10.1111/j.1399-6576.2005.00737.x 15954953

[B4] Majerus S Gill-Thwaites H Andrews K Laureys S Behavioral evaluation of consciousness in severe brain damage Prog Brain Res 2005 150 397 413 full_text 16186038 10.1016/S0079-6123(05)50028-1

[B5] Smith WD Dutton RC Smith NT Measuring the performance of anesthetic depth indicators Anesthesiology 1996 84 38 51 10.1097/00000542-199601000-00005 8572353

[B6] Kreuer S Bruhn J Larsen R Buchinger H Wilhelm W A-line, bispectral index, and estimated effect-site concentrations: a prediction of clinical end-points of anesthesia Anesth Analg 2006 102 1141 1146 10.1213/01.ane.0000202385.96653.32 16551913

[B7] Rampil IJ A primer for EEG signal processing in anesthesia Anesthesiology 1998 89 980 1002 10.1097/00000542-199810000-00023 9778016

[B8] Dahaba AA Different conditions that could result in the bispectral index indicating an incorrect hypnotic state Anesth Analg 2005 101 765 773 10.1213/01.ane.0000167269.62966.af 16115989

[B9] Schmidt GN Bischoff P Standl T Voigt M Papavero L Schulte am Esch J Narcotrend, bispectral index, and classical electroencephalogram variables during emergence from propofol/remifentanil anesthesia Anesth Analg 2002 95 1324 1330 10.1097/00000539-200211000-00042 12401620

[B10] Schmidt GN Bischoff P Standl T Lankenau G Hellstern A Hipp C Schulte am Esch J SNAP index and Bispectral index during different states of propofol/remifentanil anaesthesia Anaesthesia 2005 60 228 234 10.1111/j.1365-2044.2004.04120.x 15710006

[B11] Cortinez LI Delfino AE Fuentes R Munoz HR Performance of the cerebral state index during increasing levels of propofol anesthesia: a comparison with the bispectral index Anesth Analg 2007 104 605 610 10.1213/01.ane.0000255152.96354.17 17312217

[B12] Deogaonkar A Gupta R DeGeorgia M Sabharwal V Gopakumaran B Schubert A Provencio JJ Bispectral Index monitoring correlates with sedation scales in brain-injured patients Crit Care Med 2004 32 2403 2406 10.1097/01.CCM.0000147442.14921.A5 15599143

[B13] Fabregas N Gambus PL Valero R Carrero EJ Salvador L Zavala E Ferrer E Can bispectral index monitoring predict recovery of consciousness in patients with severe brain injury? Anesthesiology 2004 101 43 51 10.1097/00000542-200407000-00009 15220770

[B14] Brocas E Dupont H Paugam-Burtz C Servin F Mantz J Desmonts JM Bispectral index variations during tracheal suction in mechanically ventilated critically ill patients: effect of an alfentanil bolus Intensive Care Med 2002 28 211 213 10.1007/s00134-001-1189-y 11907667

[B15] Riker RR Fraser GL Sedation in the intensive care unit: refining the models and defining the questions Crit Care Med 2002 30 1661 1663 10.1097/00003246-200207000-00049 12131001

[B16] Wang Q Xu M Lei YN Wang GN Zhou JX Use of cerebral state index monitoring to detect purposeful movement in unsedated brain-injured patients J Int Med Res 2009 37 689 696 19589252 10.1177/147323000903700312

[B17] Anderson RE Jakobsson JG Cerebral state index: comparison between pairwise registrations from the left and the right sides of the brain Br J Anaesth 2006 97 347 350 10.1093/bja/ael154 16849383

